# Recent Advances in the Development of Theranostic Nanoparticles for Cardiovascular Diseases

**DOI:** 10.7150/ntno.62730

**Published:** 2021-07-21

**Authors:** Yuao Wu, Karla X. Vazquez-Prada, Yajun Liu, Andrew K. Whittaker, Run Zhang, Hang T. Ta

**Affiliations:** 1 Queensland Micro- and Nanotechnology, Griffith University, Brisbane, Queensland 4111, Australia; 2 School of Environment and Science, Griffith University, Brisbane, Queensland 4111, Australia; 3 Australian Institute for Bioengineering and Nanotechnology, the University of Queensland, Brisbane, Queensland 4072, Australia; 4 ARC Centre of Excellence in Convergent Bio-Nano Science and Technology, the University of Queensland, QLD 4072, Australia

**Keywords:** theranostics, nanomaterials, cardiovascular diseases, atherosclerosis, thrombosis, myocardial infarction, ischemic heart

## Abstract

Cardiovascular disease (CVD) is the leading cause of death worldwide. CVD includes a group of disorders of the heart and blood vessels such as myocardial infarction, ischemic heart, ischemic injury, injured arteries, thrombosis and atherosclerosis. Amongst these, atherosclerosis is the dominant cause of CVD and is an inflammatory disease of the blood vessel wall. Diagnosis and treatment of CVD remain the main challenge due to the complexity of their pathophysiology. To overcome the limitations of current treatment and diagnostic techniques, theranostic nanomaterials have emerged. The term "theranostic nanomaterials" refers to a multifunctional agent with both therapeutic and diagnostic abilities. Theranostic nanoparticles can provide imaging contrast for a diversity of techniques such as magnetic resonance imaging (MRI), positron emission tomography (PET) and computed tomography (CT). In addition, they can treat CVD using photothermal ablation and/or medication by the drugs in nanoparticles. This review discusses the latest advances in theranostic nanomaterials for the diagnosis and treatment of CVDs according to the order of disease development. MRI, CT, near-infrared spectroscopy (NIR), and fluorescence are the most widely used strategies on theranostics for CVDs detection. Different treatment methods for CVDs based on theranostic nanoparticles have also been discussed. Moreover, current problems of theranostic nanoparticles for CVDs detection and treatment and future research directions are proposed.

## 1. Introduction

Cardiovascular disease (CVD) involves a group of diseases related to blood vessels and heart dysfunction. It is one of the major causes of death and disability around the world. 1 From 2007 to 2017, the number of global deaths caused by CVD increased by 21.1% 2. The mortality of CVD before the age of 60 is constantly increasing due to risk factors of CVDs such as obesity and diabetes that tend to occur at early ages 1. Notably, the premature death rate caused by CVDs differs in countries and populations. In developing countries, the percentage of CVD-related deaths (42%) is ten times higher than in developed countries (4%)^1^. CVDs includes diseases such as atherosclerosis, thrombosis, ischaemic heart disease, coronary artery disease (CAD), cerebrovascular disease and aorta artery disease.

**Figure 1:**
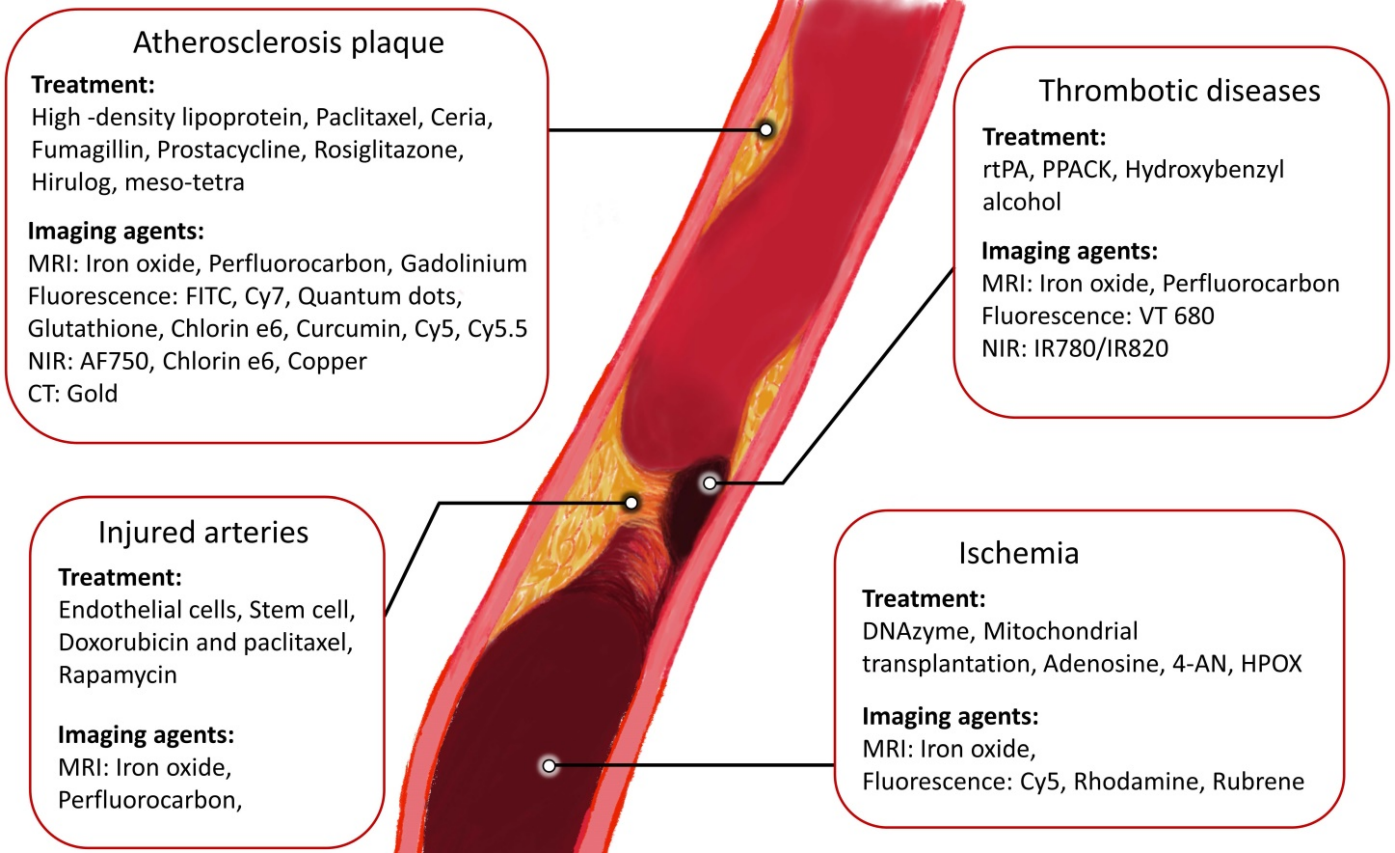
Schematic representation of the progression of atherosclerosis.

Atherosclerosis is an inflammatory disease of the wall of arteries and is the main underlying cause of CVDs 3. It is a complex disease that occurs in the intima of arteries and can be progressive for many years 4. Briefly, high levels of cholesterol and lipid accumulate in specific sites of arteries, making the inner surface of the blood vessels irregular 5. Blood flow becomes disturbed and will further contribute to the accumulation of lipids. The accumulation and deposition of both lipid and cholesterol will form an atherosclerotic plaque within the artery wall that can narrow the blood vessel. There are two major categories of plaques: stable plaques and non-stable or vulnerable plaques. Stable plaques are often asymptomatic and consist of a thick fibrous cap and a lipid core. Vulnerable plaques are rich in macrophages and possess a thin fibrous cap along with large necrotic cores with lipids. During the progression of atherosclerosis, reactive oxygen species (ROS) produced by phagocytic cells together with reactive nitrogen species (RNS) 6, 7 increase the secretion of adhesion molecules that eventually oxidize low density lipoproteins (ox-LDL) in the blood vessel. Macrophages in the vulnerable plaque will recognise and phagocitise the ox-LDL, contributing to foam cell formation. Altogether, these events will eventually rupture the vulnerable plaque due to the low collagen fibre levels in the fibrous cap and the continuous loss of vascular smooth muscle cells 8. Rupture of unstable atherosclerotic plaques and triggers the formation of blood clots and leads to thrombosis (Fig 1). Ischemia caused by thrombosis is the main factor of ischemic heart and stroke 9. In addition, there are complications to the treatment of CVDs that also worsens their progression. For instance, restenosis and vascular injury could occur due to coronary angioplasty 10, 11.

**Figure 2:**
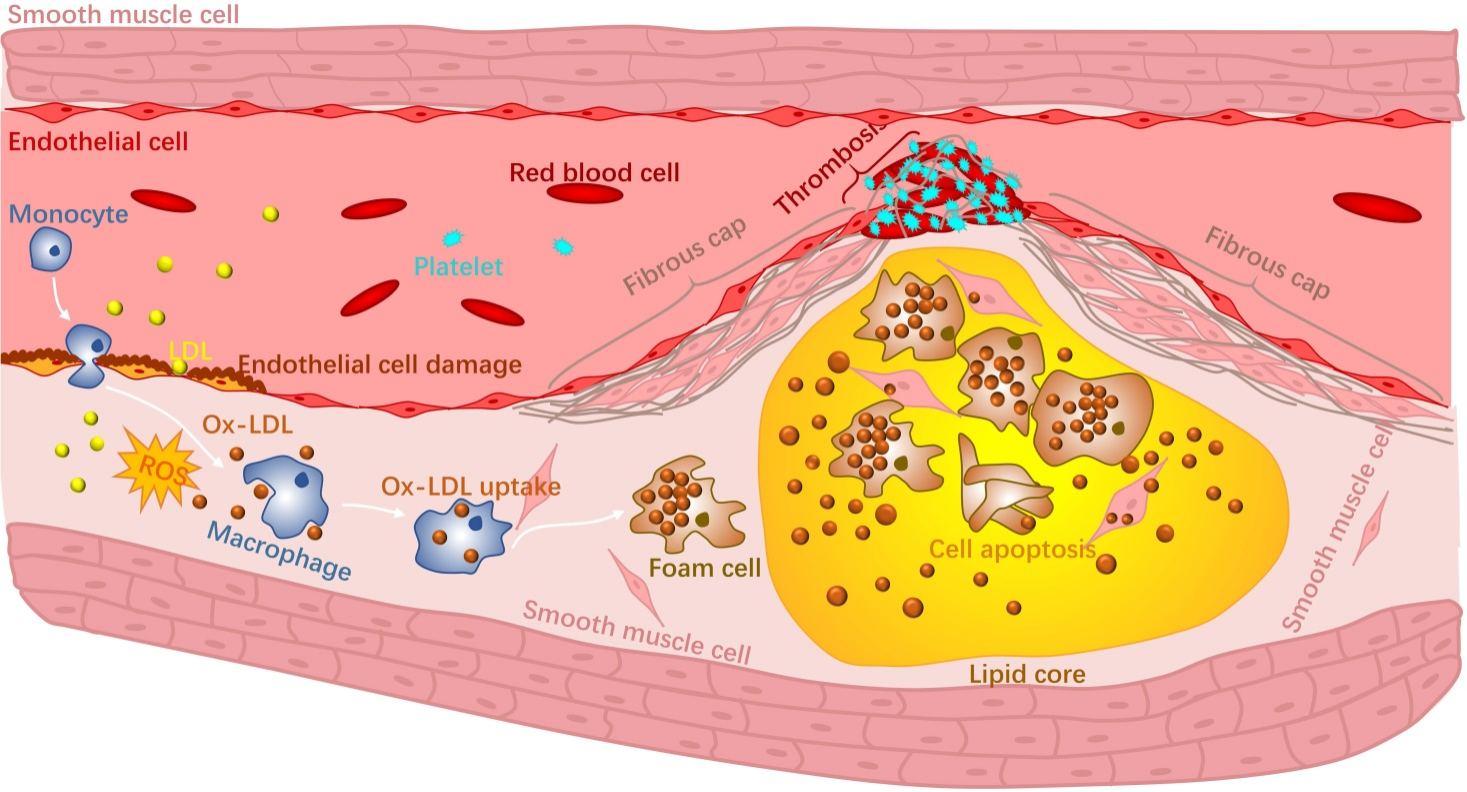
Therapeutic and imaging strategies of cardiovascular diseases.

Despite significant research progress in CVDs 12-14, they remain the leading cause of death worldwide. A variety of strategies have been applied as health care interventions 12-14. For example, multidrug therapy and aspirin are two common treatment options for people with a heart attack or stroke, which can reduce the CVDs recurrence up to 75%.^15^ With developing medical technologies, more targeted and precise strategies are needed to further improve treatment outcomes. Amongst these, theranostic materials are an emerging and promising approach that refers to the combination of therapeutic components with imaging agents (Fig 2), allowing patients to receive early detection and rapid treatment. In addition, theranostic agents are able to track drugs and monitor the therapy, making it a highly-efficient approach for targeted, safe pharmacotherapy that focuses on the patient's care. Nanobiotechnology has been a frontier and hot topic in the field of biotechnology. Several nanomaterial studies have now been applied to clinical trials and translated to be commercial products.^16^ Theranostics is a novel field of medicine that provides specific targeted therapy based on specific targeted diagnostic tests. Although current theranostic researches on CVDs are still in an immature stage, theranostic nanoparticles have developed rapidly in the last decades due to the advantages of combining diagnosis and treatment in one single agent. There are a few reviews discussing the development of the diagnostic or therapeutic nanoparticles only for atherosclerosis 17, 18. In this review, we collate and discuss different current existing theranostic nanomaterials, including the latest advances in the last 3 years, for different cardiovascular diseases and conditions including atherosclerosis, thrombosis, injured arteries and ischemia. The therapeutic, imaging and targeting strategies are reviewed. Clinical translational possibility and current problems of theranostic nanoparticles for CVDs are discussed. Finally, future research directions are proposed.

## 2. Theranostic nanoparticles for atherosclerosis

As atherosclerosis is the leading cause of cardiovascular-related death, multiple theranostic nanomaterials have been developed for the treatment of atherosclerosis in the last decades.^4^ Atherosclerosis is the first step in the progression of many CVDs. When a vulnerable atherosclerotic plaque ruptures, thrombogenic material can be released and form clots in the blood vessels, which can lead to myocardial infarction (MI), stroke and ischemic injuries.^4^, ^19^ In this section, the MRI, CT, NIR and fluorescence imaging agents for prudential detection of atherosclerosis are discussed and summarised in Table 1.

### 2.1 MRI based theranostic nanoparticles for atherosclerosis

Nandwana et al. 20 coated phospholipids and ApoA1 onto iron oxide magnetic nanostructures (HDL-MNS) to mimic natural HDL for detection, prevention and treatment of atherosclerosis. The formation of atherosclerotic plaques is caused by the accumulation of cholesterol and lipids, which are eventually phagocytised by macrophages.^8^ Macrophages with subsequent accumulation of low-density lipoproteins (LDL) will form foam cells, causing fatty streak and early atherogenesis.^21^ High-density lipoproteins (HDL) expel and carry cholesterol from peripheral lipid-laden macrophages to the liver, a process known as reverse cholesterol transport (RCT). This process can cause the plaques either to regress or grow slowly 22-25. HDLs are composed of an outer apolipoprotein (Apo) and inner cholesterol ester. Their high-density lipoprotein-like magnetic nanostructures (HDL-MNS) have proven to be an effective MRI contrast agent, as it increased the T_2_-weighted dark signal (r_2_ = 383 mM^-1^, s^-1^, at 7 T) five-fold compared with Ferumoxytol (commercial iron MRI contrast agent). In terms of treatment, HDL-MNS particles showed cholesterol efflux values of 4.8% with a similar ability to excrete cholesterols compared with natural HDL (4.7%). Oumzil et al.^26^ loaded prostacyclin into solid lipid nanoparticles (SLNs) together with ultrasmall superparamagnetic iron oxide (USPIO) particles for image-guided therapy. In platelet aggregation studies, SLNs loaded with prostacyclin completely inhibited platelet aggregation after 3 hours and 15 minutes of treatment. This particle also showed a 2.6-fold higher MRI signal (r_2_ = 557 mM^-1^, s^-1^) at 4.7 T compared to the clinical superparamagnetic iron oxide contrast agent Feridex^®^.

Recently, Ta group developed cerium oxide-coated iron oxide (IO@CO) nanoparticles^26^ and chitosan nano-cocktails containing both cerium oxide and iron oxide (Chit-IOCO) for ROS clearance in inflamed macrophages.^27^, ^28^ Chitosan, a linear polysaccharide composed of randomly distributed β--linked D-glucosamine and N-acetyl-D-glucosamine 29, was used as a carrier. Cerium oxide was employed as it has regenerative anti-ROS ability when cerium switches between trivalence and tetravalence states 30, 31. *In vitro* studies indicated that the nanoparticles could significantly decrease the ROS level in activated macrophages. Moreover, cell MRI studies showed that both IO@CO (r_2_ = 339 mM^-1^, s^-1^, at 9.4 T) and Chit-IOCO (r_2_ = 308 mM^-1^, s^-1^, at 9.4 T) were promising contrast agents for MRI. According to our results, this nanoparticle can be considered as a potential theranostic material for ROS-related inflammatory diseases. In addition, we also synthesised CeO_2_-Fe_3_O_4_@LDH nanocomposites 32. The rationale of these nanocomposites is similar to the above nanoparticles. Layered double hydroxide (LDH) was used as a carrier 33 to load both iron oxide and cerium oxide nanoparticles. This multifunctional nanosystem not only provided excellent MRI signals (r_2_ = 243 mM^-1^, s^-1^, at 9.4 T) in the macrophages, but also quenched the intracellular ROS after H_2_O_2_-stimulation.

Besides iron oxide nanoparticles, paramagnetic perfluorocarbon nanoparticles conjugated with fumagillin were used for the treatment and imaging of atherosclerosis in another study. Specific delivery of the antiangiogenic drug and enhanced MRI contrast were observed 34. *In vivo* MRI tests showed a significantly decreased MRI signal in the atherosclerotic plaques of cholesterol-fed rabbits after one week of injection of the drug-loaded nanoparticles. The nanoparticles that were not loaded with the drugs did not show any changes in MRI intensity post-injection. These results indicated that the nanosystem could be a potential theranostic agent for atherosclerosis.

Given recent developments, MRI is the most promising strategy diagnosis method of atherosclerosis, as it showed high spatial resolution images in the body and is a no radiation involved-technology. Iron oxide nanoparticles are the most widely MRI agents used in cardiovascular diseases 35-51 in general and specifically in theranostic nanoparticles for the diagnosis of atherosclerosis. There has been no theranostic material based on gadolinium developed for atherosclerosis. Most preclinical iron oxide nanoparticles showed less toxicity than gadolinium MRI agents 52, 53. Moreover, better MRI contrast ability of most preclinical iron oxide nanoparticles can be observed, compared to the commercial iron oxide MRI contrast agents such as Feridex® (r_2_=307 at 9.4T, r_2_=93 at 3T), FeREX® (r_2_=284 at 9.4T, r_2_=160 at 3T) and Resovist® (r_2_=143 at 3T) 54. Iron oxide nanoparticle conjugated with antibodies showed their potential ability to image atherosclerosis plaques. Paramagnetic perfluorocarbon nanoparticles were also a non-toxic MRI agent and can avoid background noise. However, the minimum detectable concentration of ^19^F in the target place is higher than iron oxide; hence the MRI sensitivity of paramagnetic perfluorocarbon nanoparticles is lower than iron oxide nanoparticles. Overall, iron oxide nanoparticles will still be the main research direction of MRI imaging in the future.

### 2.2 Fluorescence based theranostic nanoparticles for atherosclerosis

**Figure 3:**
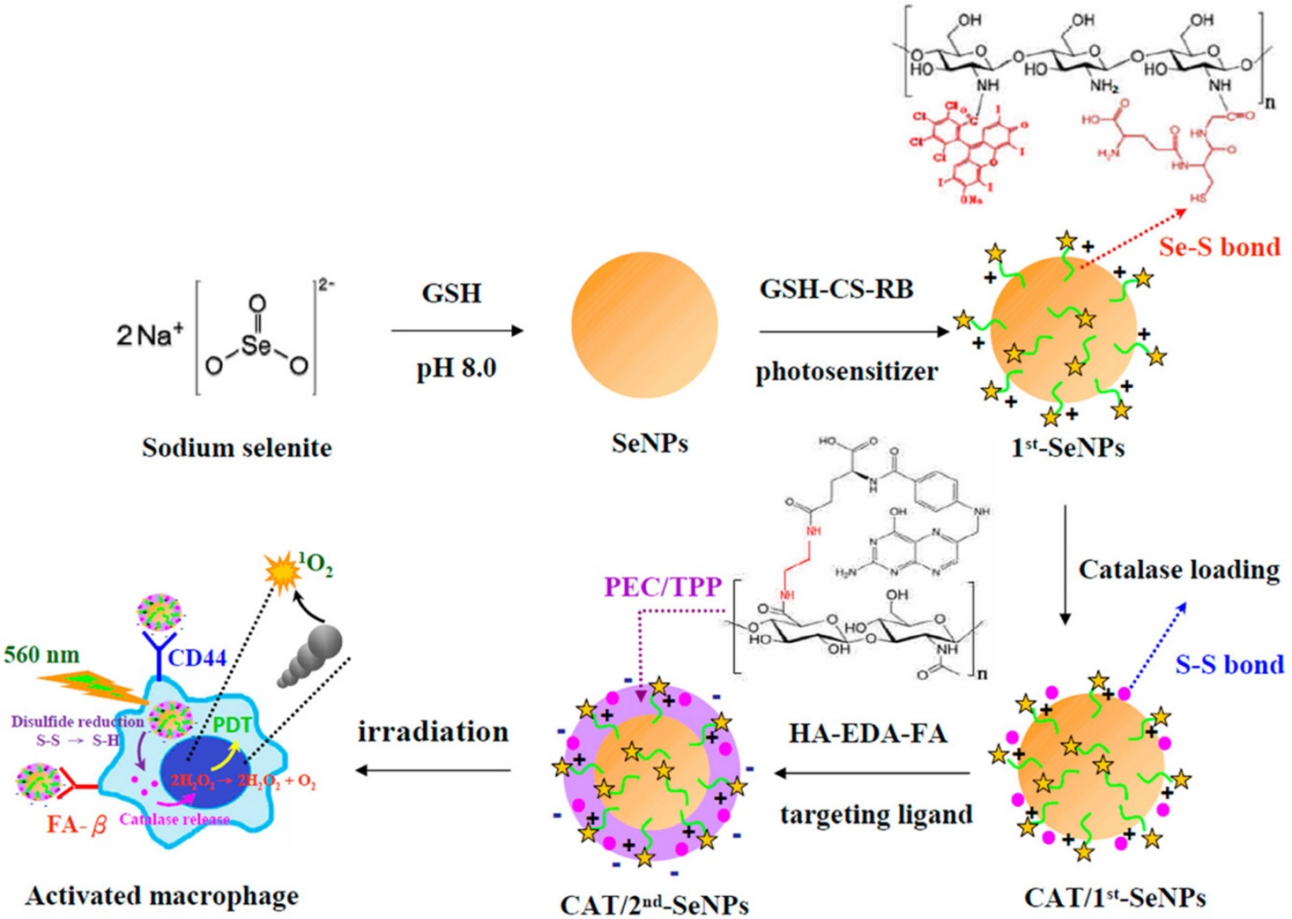
Schematic diagrams for the preparation of catalase-loaded SeNPs.^56^

In addition to MRI, fluorescent agents are also commonly used to locate and detect atherosclerosis lesions. In atherosclerosis, inflamed macrophages promote the formation of plaques, increase the core of necrosis and weaken the fibrous cap. Therefore, treatments that target inflamed macrophages could also be a feasible strategy for atherosclerosis.^4^, ^55^ Recent study has shown that activating nanomaterials with near-infrared light to treat inflammatory macrophages is feasible. Lu et al.^56^ developed photodynamic selenium nanoparticles (SeNPs) targeting inflammatory macrophages. These multiple layered spherical nanoparticles had selenium nanoparticles at the centre. Firstly, selenium (Se) nanoparticles were coated by RB-CS-GSH, chitosan (CS) conjugated with rose bengal (RB, the photosensitizer) and glutathione (GSH), via Se-S bonds. Then catalases were conjugated onto the nanoparticles via disulphide bonds with RB-CS-GSH to form the first coating layer. Selenium nanoparticles were employed to quench H_2_O_2_ in the inflammatory macrophages. RB-CS-GSH layer together with catalases was used for imaging and photodynamic therapy. Secondary, they covalently conjugated the carboxyl groups from both hyaluronic acid (HA) and folic acid (FA) with the amine groups from ethylenediamine (EDA) to form HA-EDA-FA mixture. Subsequently, negatively charged HA-EDA-FA was loaded onto the positively charged RB-CS-GSH via electrostatic interactions as the second layer of the nanoparticles. HA and FA was employed as binding ligands to folate receptor beta (FR-β) and CD44 on the surface of the inflammatory macrophages (Fig 3). *In vitro* studies indicated that, compared to non-stimulated macrophages, SeNPs provided stronger fluorescence signals of RB in lipopolysaccharide (LPS)-stimulated macrophages. It indicated that SeNPs were able to specifically bind to active macrophages via the folic acid and HA coating. Phototoxicity studies showed that SeNPs could kill activated macrophages as catalases transforming H_2_O_2_ to the toxic singlet oxygen ^1^O_2_. At the same time, The H_2_O_2_ level was effectively reduced (85.2%) in LPS-stimulated macrophages by transferring H_2_O_2_ to the toxic singlet oxygen ^1^O_2_. As a result, both the amount of activated macrophage and the released hydrogen peroxide and NO levels were reduced in the lesions, thereby inhibiting the development of inflammation. Non-active macrophages were not damaged by these nanoparticles. The results showed potential therapeutic and imaging applications of these nanoparticles.

In another study of macrophage-targeted theranostic nanoparticles, Yi et al.^57^ loaded chlorin e6 (Ce6) as a photosensitizer in their dextran sulphate-deoxycholic acid (DS-DOCA) nanomaterials. Cytotoxicity tested by methyl thiazolyl tetrazolium (MTT) indicated that Ce6/DS-DOCA-treated macrophage cells showed approximately 80% of cell deaths when compared to the non-treated macrophage (20%) after 2 minutes of laser irradiation. Although the imaging performance of the material was not mentioned in the text, according to the nanomaterials above, we can speculate that DS-DOCA nanomaterials would be a potential NIR imaging agents based on fluorescence emission from the photosensitizers.

Hou et al.^58^ loaded curcumin (Cur) into oligomeric hyaluronic acid-2′-[propane-2,2-diyllbls (thio)] diacetic acl-hydroxymethylferrocene (oHA-TKL-Fc) to form nanogels (HASF@Cur) by the self-assembly method for reducing the ROS inside macrophages. Fluorescence images of HASF@Cur indicated that HASF@Cur more readily aggregated in the macrophages than free Cur. In addition, *in vivo* studies showed that HASF@Cur had fewer lesions in atherosclerotic rat models when compared with the free curcumin. The nanoparticles were detected by the fluorescence of curcumin. This study indicated that that curcumin could be used as an anti-ROS drug and also an imaging substance. However, the mechanism of curcumin acting on the macrophages has not been well explained in the current studies.

Kosuge et al. 59 designed a single-walled carbon nanotube (SWNT) functionalized with Cy5.5 dye that could be used for near-infrared (NIR) imaging and photothermal ablation of inflammatory macrophages. *Ex vivo* NIR imaging showed a strong signal in ligated left carotid arteries in mice treated with SWNTs while the ones without SWNT treatment showed no NIR signal. Confocal microscopy and *ex vivo* thermal ablation experiments indicated that SWNTs could effectively induce apoptosis of macrophages. In general, SWNTs have proven to be a theranostic nanomaterial capable of fluorescence imaging and photothermal ablation of vascular macrophages.

Marrache et al. 60 developed a synthetic HDL nanoparticle composed of triphenylphosphonium (TPP), apolipoprotein (Apo) A-I mimetic 4F(four phenylalanine residues) peptide and Qdot® 705 ITK™ amino PEG quantum dots core (QDs) for the diagnosis and treatment of atherosclerosis. Experiments showed that these HDL nanoparticles have a similar cholesterol binding affinity as endogenous HDL. The nanomaterials showed excellent targeting, biocompatibility and ability to degrade lipids. Similarly, Sun et al.^61^ developed a novel trifunctional virus-like simian virus 40 (SV40)-based nanoparticle to deliver Hirulog peptide. The nanosystem was also loaded with near-infrared quantum dots and labelled with cyclic peptides (CGNKRTRGC) for targeting p32 protein on macrophages. Targeted nanoparticles provided 3-fold stronger fluorescence signal in the plaques than non-targeted nanoparticles in ApoE(-/-) mice. The resulting SV40 nanoparticles were able to selectively deliver Hirulog to atherosclerotic plaques in ApoE knockout mice. Moreover, stronger antithrombin activity was detected in the aortas of mice injected with SV40 nanoparticles than the non-treated mice. This new type of nanoparticle will be suitable for molecular targeting, *in vivo* imaging, and drug delivery in atherosclerosis. Peters et al. 62 developed carboxyfluorescein (also known as FAM™)-PEG micelles that were labelled with cysteine-arginine-glutamic acid-lysine-alanine (CREKA) and also loaded with Hirulog for treatment of atherosclerotic plaques. CREKA is fibrin targeting peptide and Hirulog is a thrombin inhibitor peptide. The results showed that CREKA-targeted micelles had stronger antithrombotic properties than non-targeted nanomicelles in ApoE knockout mice.

Fluorescent agents are mainly used to track the cellular uptake and biodistribution of nanomaterials. Results showed clear fluorescence images on *in vitro, ex vivo* and *in vivo* experiments. Unfortunately, fluorescent agents are unlikely to transfer to clinical trials as the background fluorescence of human body is much higher than in the cell or mice. Therefore, using fluorescent agents in theranostic nanoparticles have no advantage in future clinical diagnosis.

### 2.3 Computed tomography (CT) based theranostic nanoparticles for atherosclerosis

Precious metal nanostructured gold was employed as computed tomography (CT) contrast agent and photothermal (PTT) agent for theranostics of cardiovascular diseases 63-65. Qin et al. 66 synthesised gold nanorods (Au-NRs) as a platform for theranostics of inflammatory macrophages. Micro-CT imaging of macrophages demonstrated that the signal intensity increased in a concentration-dependent manner. *In vivo* thermal therapy in Apo E knockout mice indicated a slight enhancement of CT intensity in the inflamed femoral artery after intravenous injection of Au-NRs. Besides, a significant increase of temperature at the inflamed femoral artery (up to 50.5 °C) was detected under the laser treatment (808 nm). CD68-stained histological results showed that an obvious macrophage damage occurred in the femoral artery restenosis. This nanosystem has proven to be non-toxic and promising as a new theranostic platform for atherosclerosis. Computed tomography agents such as gold showed the potential ability for the diagnosis of atherosclerosis. Gold nanoparticles also show a higher contrast-to-noise ratio (CNR) than the current commercial iodinated agent Visipaque™ 67. However, CT imaging technology itself has radiation exposure and it is unlikely to perform multiple scans in a short period of time clinically. In the future, more sensitive CT agents may reduce the amount of radiation needed to reduce radiation-related damage.

### 2.4 Photoacoustic based theranostic nanoparticles for atherosclerosis

A study suggested that copper sulphide nanoparticles (CuS) could be a feasible photoacoustic agent for the diagnosis and treatment of atherosclerosis.^68^. CuS nanomaterial was conjugated with monoclonal antibody targeting transient receptor potential cation channel subfamily V member 1(TRPV1). The nanoparticles caused a temperature increase in smooth muscle cells on NIR laser irradiation. The increase in temperature activated TRPV1 cation channels to reduce lipid accumulation and foam cell formation, which eventually alleviated the conditions of atherosclerosis. At the same time, due to the illumination with the NIR laser, a strong photoacoustic signal was generated in the target areas and was used for optical imaging of atherosclerosis. CuS-TRPV1 was able to provide a significant photoacoustic signal of the vascular structure of the heart. After 12 weeks of intravenous injection of the nanomaterials, lipid storage and atherosclerotic lesions in the aorta of Apo E knockout mice were significantly reduced. Histological analysis showed no significant organ damage. Photoacoustic imaging is another strategy for the diagnosis of atherosclerotic lesions. It is a real-time technology that can be applied in clinical trials. Some accompanying heat effects could also improve the treatment of atherosclerosis. However, the sensitivity and resolution of photoacoustic imaging still need to be improved.

### 2.5 Multiagent based theranostic nanoparticles for atherosclerosis

McCarthy et al. 69, 70 synthesised magnetic fluorescent nanoparticles for theranostics of inflammatory atherosclerosis. Dextran-coated iron oxide nanomaterials were modified using AlexaFluor 750 for near-infrared fluorescence and meso-tetra(m-hydroxyphenyl) chlorin (THPC) for phototoxic treatment of inflammatory macrophages. *In vivo* near-infrared (NIR) imaging experiments in C57/BL6 mice have shown that THPC-based nanomaterials could specifically aggregate in areas with abundant macrophages and foam cells. After exposure to 650 nm wavelength laser light, the nanomaterial significantly reduced the plaque areas. This study suggested that this therapeutic nanomaterial was feasible for the treatment of atherosclerotic vascular diseases. The authors also developed novel hydrophilic photosensitizers based on 5‐(4‐carboxyphenyl)‐10,15,20‐triphenyl‐2,3‐dihydroxychlorin (TPC) and magnetic nanoparticles that could treat atherosclerosis by inducing the death of macrophages. *In vitro* experiments indicated that human macrophages could reach up to 100% death rate after treated with TPC nanoparticles for 60 minutes followed by 3 minutes exposure of 650 nm laser light. The macrophages treated with magnetic nanoparticles without TPC did not show cytotoxicity. The MRI and fluorescence images nanoparticle solution phantoms indicated these nanoparticles have potential capability to be functionalized as the diagnostic nanosystem. The imaging capability of the nanoparticles has not been demonstrated *in vitro* and *in vivo* to this time.

Bagalkot et al. 71 synthesised a lipid-latex (LiLa) nanoparticles with phosphatidylserine and oxidized cholesterol ester derivative cholesterol-9-carboxynonanoate that could selectively target macrophages of the M1 inflammatory phenotype. This nanosystem also included gadolinium (Gd) and fluorescein isothiocyanate (FITC) to implement MRI detection and optical screening. The combined nanoparticles Gd-FITC-LiLa allowed non-invasive MR imaging of atherosclerotic plaques in ApoE^-/-^ mice. They could also be selectively deposited into macrophages in inflamed adipose tissues and served as a drug carrier. Rosiglitazone (Rosi) was loaded in this nanocarrier to provide an anti-inflammatory therapeutic effect. Results suggested that LiLa nanoparticles are a novel theranostic nanosystem that could potentially be used in atherosclerosis imaging and therapy.

Overall, using theranostic nanomaterials is an effective way for the treatment and diagnosis of atherosclerosis. Iron oxide-based MRI showed low toxicity and excellent MRI contrast ability. It is also considered to be the most promising diagnostic strategy for atherosclerosis. If the cost and the detection time of MRI can be reduced, iron oxide-based MRI will be the most mainstream diagnostic method for atherosclerosis in the next decades. Many nanoparticles loaded with chemicals or drugs showed better treatment effects than the free form. It is worth to synthesise the current drugs on the nanoscale to improve their therapeutic effect on atherosclerosis. Additionally, antibodies or polypeptides modification will increase the specific binding of theranostic nanoparticles to atherosclerotic lesions, resulting in more efficient treatment effects and stronger imaging abilities than non-targeted nanoparticles. Vitronectin, CD44, and dextran receptors on macrophages are the popular biomarkers used for targeting. It is recommended to use targeting strategies for future theranostic nanomaterials.

## 3. Theranostic nanoparticles for thrombosis

Thrombus, known as blood clot, is a solid mass of coagulated blood. It is usually caused by the rupture of unstable atherosclerosis plaques. Pathological arterial thrombosis is formed by complex interactions between environmental and genetic factors 72. Thrombosis is the major cause of many CVDs 19, 73. rtPAs are widely used as antithrombotic drugs. rtPA is a recombinant protein that participates in the breakdown of blood clots. Like natural tissue plasminogen activator (tPA), it acts as an enzyme that catalyses the conversion of plasminogen to plasmin and serine proteases that lyse the blood clots. Compared to natural tPA, rtPA has a longer half-life 74-76.

**Figure 4:**
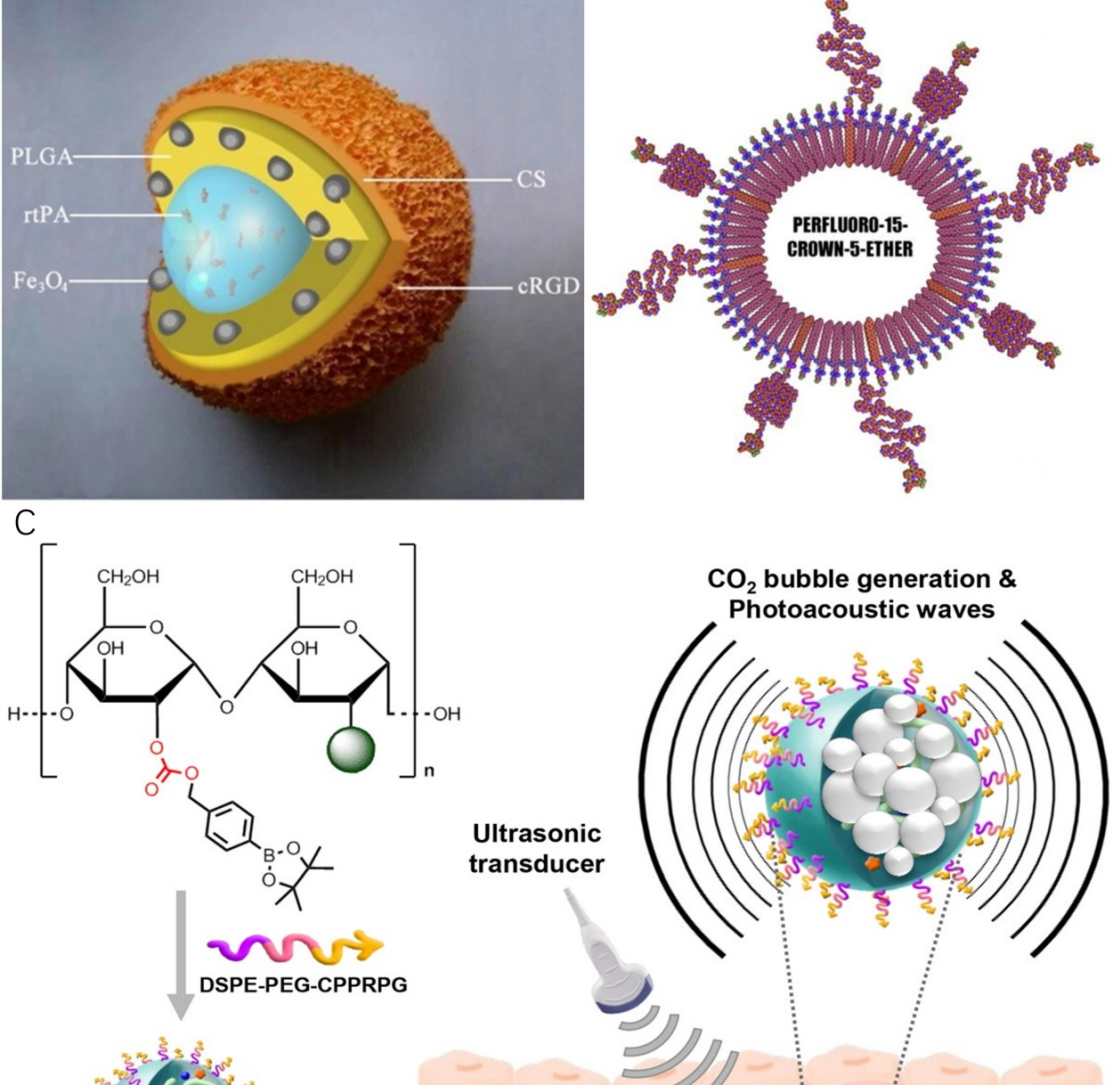
Theranostic nanoparticles for thrombosis. **(A)** Schematic representation of an Fe3O4-PLGA-rtPA/CS-cRGD nanoparticle.^79^ **(B)** Schematic of the PPACK-functionalized PFC-core nanoparticle^88^ **(C)** Schematic illustration of T-FBM nanoparticles as a thrombus-specific nanotheranostic agent. T-FBM nanoparticles target a fibrin-rich thrombus and serve as a H_2_O_2_-triggered photoacoustic signal amplifier but also an antithrombotic nanomedicine.^91^

In 2009, Ma et al 77 synthesised a polyacrylic acid-coated magnetic nanoparticle conjugated with rtPA for thrombolysis and diagnosis of thrombosis (PAA-MNP-rtPA). Studies showed that administration of 0.2 mg/kg of PAA-MNP-rtPA could effectively dissolve the thrombus in rat right iliac arteries at 25 minutes by magnetic guidance, whereas 0.5 mg/kg of free rtPA showed no significant evidence of thrombolysis. In this study, no MRI was performed. Due to the iron core of the nanosystem, it might be worth doing follow-up studies testing PAA-MNP-rtPA as contrast agents for MRI. A similar nanoparticle was developed by Yang et al. 78 who synthesised a core-shell nanoparticle with poly [aniline-co-N-(1-one-butyric acid) aniline] shell and Fe_3_O_4_ magnetic nanoparticle core for thrombolysis. The nanoparticles carried 276 μg of active rtPA per mg for the breakdown of blood clots. Clot dissolution time was reduced from 39.2 ± 3.2 to 10.8 ± 4.2 minutes by magnetic guidance. The biodistribution of this nanosystem in rats was evaluated by single-photon emission computed tomography (SPECT)/computed tomography (CT). In another research, Zhou et al. 79 synthesised a multifunctional Fe_3_O_4_-based PLGA nanoparticle with a core of rtPA and shell of Fe_3_O_4_ and poly(lactic-co-glycolic acid) (PLGA) as well as a cyclic RGD (cRGD)-chitosan (CS) membrane (Fe_3_O_4_-PLGA-rtPA/CS-cRGD)(Fig 4A). Compared to free rtPA, Fe3O4-PLGA-rtPA/CS-cRGD nanoparticles dissolved the clots to a three-fold greater extent at 60 minutes. MRI studies showed that the CS-cRGD coating did not affect the MRI signal, and the nanoparticles showed a strong T_2_ signal in the rat abdominal aorta containing thrombus. Effective treatment and imaging largely depended on the specific targeting function of Arg-Gly-Asp (RGD) peptides. As a receptor antagonist of the platelet membrane glycoprotein GP IIb/IIIa, RGD could bind to activated platelets at the thrombus sites 80. In another study, bubble liposomes (BLs) conjugated with RGD peptides were synthesised for theranostics of thrombosis.^81^ This nanoparticle showed a significant enhancement of thrombolysis *in vivo* with a low-frequency and high-intensity transcutaneous ultrasound exposure. A shorter average reperfusion time (16.7 ± 5.0 min) in rabbits was observed to achieve thrombolysis in myocardial infarction (TIMI) grade 3 flow (a coronary blood flow grading scale system that visually evaluates the opacity velocity of infarct arteriography) in comparison with the non-targeted nanoparticles (60.0 ± 0 min) and free rtPA (41.3 ± 14.4 min). Together with ultrasound imaging capability, the (BLs)-RGD nanoparticle are expected to be a novel non-invasive echo contrast agent and recanalisation therapy for thrombosis.

Besides rtPAs, blood transglutaminase FXIIIa is another useful biomarker for acute thrombosis 82. FXIIIa crosslinks fibrin chains to stabilise newly formed thrombi. It also incorporates alpha 2 anti-plasmin into the clot to increase fibrinolysis resistance 83, 84. McCarthy et al. 85 conjugated an anti-FXIIIa peptide (GNQEQVSPLTLLKC), rtPA and fluorophore to the crosslinked dextran-coated iron oxide (CLIO) for thrombus targeting, fibrinolytic therapy and imaging respectively. The results indicated that CLIO-FXIII-PEG-rtPA-VT680 nanoparticles had significant outcomes for thrombolysis in mice compared to free rtPAs. MRI results suggested that CLIO-FXIII-PEG-rtPA-VT680 was a good MRI contrast agent.

An alternative to rtPA is D-phenylalanyl-l-prolyl-l-arginyl-chloromethyl ketone (PPACK). PPACK is a highly potent irreversible thrombin inhibitor with a strong affinity for thrombin 86, 87. Myerson et al. in 2011^88^, covalently immobilised PPACK on the surface of a perfluorocarbon nanoparticle structure for the treatment and MR imaging of acute thrombosis (Fig 4B). The results confirmed that the ability of PPACK nanoparticles (160 nm) to inhibit thrombus formation was significantly higher than heparin and free PPACK. In addition, PPACK nanoparticles could specifically bind to acute thrombotic lesions and enable detection of the thrombus by MRI. In a follow-up study, Palekar et al. 89, 90 found that with the accumulation of thrombin in the coronary stent, PPACK nanoparticles continuously bound to and inactivated thrombin molecules on the surface, forming an "anti-coagulation" surface. Results showed that these PPACK perfluorocarbon nanoparticles were also able to prevent the formation of coronary stent thrombosis.

Recently, Jung et al. 91 designed thrombus-specific (GPRPP- pentapeptide decorated) near-infrared fluorescent-dye-conjugated boronated maltodextrin (T-FBM) theranostic nanoparticles (Fig 4C). The system was activated by H_2_O_2_ produced during thrombosis. H_2_O_2_ reacted with the borylbenzyl carbonate of boronated maltodextrin to generate carbon dioxide bubbles, which enhanced the signal of ultrasound/photoacoustic imaging. *In vitro* studies showed that T-FBM nanoparticles could significantly inhibit ROS production in H_2_O_2_-stimulated cells and inhibit the expression of the pro-inflammatory protein (TNF-α and IL-1β) by releasing of hydroxybenzyl alcohol (HBA). In addition, lower CD40 ligand levels in T-FBM treated platelet-rich plasma indicated that these nanoparticles could also significantly suppress the activity of the platelets. *In vivo* studies showed that T-FBM could target fibrin-rich thrombi well as fluorescence intensity significantly increased from 5 minutes to 30 minutes post-injection in mice with FeCl_3_-induced injured arteries. The expression of TNF-α and sCD40L in the carotid artery and blood of the mice was detected by ELISA. Results indicated T-FBM suppressed the level of TNF-α and sCD40L in FeCl_3_- treated mice. This nanosystem is a promising theranostic platform for H_2_O_2_-related CVDs. The same group designed a similar theranostic nanomaterial for targeting fibrin. This fibrin-targeted imaging and antithrombotic nanomedicine (FTIAN) 92 enhanced photoacoustic imaging of the thrombus in the mouse models. It also showed promising results *in vivo* based on both anti-inflammatory and anti-platelet activity experiments.

Table 2 summarises the theranostic nanomaterials developed for thrombosis. In all, theranostic nanomaterials work effectively in the treatment and diagnosis of thrombosis. RtPAs are the most effective and widely used in the treatment of thrombosis. MRI, NIR and fluorescent agents were used for the imaging of the thrombosis. Still, iron oxide-based MRI are the mainstream strategy for strong and sensitive imaging. Moreover, peptides conjugation can significantly increase the aggregation of the nanoparticles at the thrombi.

## 4. Theranostic nanomaterials for myocardial infarction (MI), ischemic heart disease and ischemic injury

Myocardial infarction (MI) is a heart muscle injury caused by the blockage of blood supply to the heart, and is mainly due to the thrombosis. Around 60-80% of MI caused by thrombosis is triggered by the rupture of the vulnerable atherosclerotic plaques. The remaining 20%-40% is caused by erosion of the intimal surface 41, 42. MI can have serious consequences such as arrhythmia, heart failure, cardiogenic shock or cardiac arrest, leading to morbidity and mortality 93, 94. Some of theranostic nanomaterials developed for MI, ischemic heart disease and ischemic injury were discussed in this part (Table 3).

The pro-inflammatory cytokine tumour necrosis factor-α (TNF-α) is considered to be responsible for irreversible damage to the heart during the onset of MI.^95^, ^96^ The pathogenesis of TNF-α-mediated MI is triggered by a combination of factors, including initiation of the proinflammatory cytokine cascades and induction of ROS and RNS 95, 97. Therefore, effective TNF-α inhibition during the inflammatory phase is an important strategy for the treatment of MI. Oligonucleotide-based gene silencing technology has been applied to inhibit the expression of TNF-α gene 98-100. Somasuntharam et al. 101 synthesised Cy5-labelled-deoxyribozyme (DNAzyme)-functionalized gold nanoparticles (AuNPs) for MI treatment. They knocked down TNF-α gene expression by DNAzyme with TNF-α mRNA in macrophage RAW 264.7 and tested the nanoparticles in adult Sprague Dawley rats. These nanoparticles showed a significant knocking down of TNF-α expression in LPS-stimulated macrophages. Besides, *ex-vivo* fluorescence images of the mice heart demonstrated that the nanoparticles could be detected at least up to 3 days after injection. Overall, the above gold nanoparticles exhibit their potential to be theranostic drugs.

Ischemic heart disease is the earliest stage of MI. Initially, atherosclerotic plaque induces stenosis of coronary artery, resulting in reduced blood flow and insufficient myocardial blood supply. As the disease progresses, plaque can rupture, inducing thrombosis and eventually leads to occlusion.^4^, ^102^ treatment of early ischemic heart diseases might provide a good strategy for the treatment of MI 93, 103, 104. Feiner et al. 105 developed a novel hybrid cardiac patch in the combination of heart cells with flexible stand-alone electronics and 3D nanocomposite scaffolds for the early detection of ischemic heart diseases. In this system, a scaffold consisting of a compact nanofiber network was combined with an electronic component. Heart cells were subsequently incubated into the hybrid to finalise the engineered tissue. The synthetic tissue was then folded to make a thick, self-contained microelectronic heart patch. The patches contained multiple electrodes to record tissue functions and detect early warning of heart disorder. These electrodes also improved the function of the engineered tissue and its surroundings, regarding sensing, stimulation and regulation. The electronic film can allow tissue growth between the electrodes. Experiments showed that the hybrid cardiac patches could record cellular electrical activity and provide electrical stimulation to synchronise cell shrinkage. The deposition of an electroactive polymer on a specific electrode allows for effective, on-demand, controlled release of the drug.

Cheng et al. 106 used stem cell transplantation technology as a therapeutic strategy for cardiac regeneration after myocardial infarction. Iron oxide nanoparticles were used as a cell-ligation mediator by modifying with anti-CD34 and anti-CD45 on the surface. The nanoparticles worked as magnetic bifunctional cell engagers (MagBICE) to connect exogenous bone marrow-derived stem cells (recognised by anti-CD45) and endogenous injured cardiomyocytes (recognised by anti-CD34). The study showed that these iron oxide nanoparticles could be enriched at the damaged myocardium and visualised by magnetic resonance imaging (MRI) when MagBICE were injected intravenously into the rats. Furthermore, stem cells were delivered more efficiently to the target location by magnetic attraction. Studies of therapeutic effects have shown that there were more cardiomyocytes in the area around the heart infarction treated by MagBICE nanomaterials compared to normal cell transplantation in female Wistar-Kyoto rats by 3 h ischemia and 20 min reperfusion.

Cowan et al. 107 developed an approach using labelled mitochondria for cardioprotection. Mitochondria was labelled with fluorine-18-rhodamine 6G and iron oxide nanoparticles to allow detection using positron emission tomography (PET), fluorescence imaging, magnetic resonance imaging (MRI) and microcomputed tomography (μCT). Transmission electron microscopy and fluorescence images indicated that iron oxide nanoparticles and Fluorine-18-rhodamine 6G could successfully attach to amine groups on mitochondria surface as expected. Mitochondrial transplantation was used at the ischemic areas to increase adenosine triphosphate (ATP) content in the tissues in order to reduce infarct size. Tests of myocardial function of the rabbit ischemic hearts showed a significant reduction in infarct size after treatment with these labelled mitochondrias. ^18^F-R6G-labeled mitochondria provided a clear PET signal and T_2_-weighted MRI signal in the ischemic left ventricle. In the normal right ventricle, the MRI and PET signals did not appear. This technique could reduce cell loss, enhance post-ischemic contractile function and reduce infarct size to protect the heart 108, 109. It showed a significant reduction in infarct size in mitochondria-transplanted hearts than the normal hearts control.

Lee et al. 110 synthesised hydrogen peroxide-responsive copolyoxalate nanoparticles for ischemic injury. In this study, hydroxybenzyl alcohol (HBA) was conjugated with copolyoxalate nanoparticles (HPOX) and loaded with rubrene (Rb) as a fluorophore. HBA is one of the main active pharmaceutical ingredients of Gastrodia elata and has excellent anti-inflammatory activity. HBA in these HPOX nanoparticles could effectively eliminate hydrogen peroxide produced from ischemic injury because HPOX could specifically react with the H_2_O_2_. Furthermore, *in vivo* chemiluminescent images demonstrated that Rb-labelled nanoparticles HPOX/RB could reduce the H_2_O_2_ levels in the injured areas of mice. In a mouse hind limb I/R model, *in vivo* images of the damaged areas showed a strong fluorescence intensity 2 minutes after the injection of the HPOX/RB. In the model without hydrogen peroxide, the detected fluorescence was significantly lower. This demonstrates that HPOX/RB nanoparticles are able to detect the hydrogen peroxide released by the damaged tissues, hence locate the internal injured sites.

Generally, biological agents such as enzymes, organelle and stem cells are used for recovering the damage caused by ischemic diseases. Only mitochondrial transplantation and stem cell transplantation technology showed the direct reduction of infarct size. Hence, cells or organelle transplantation could be the trends in future treatment strategies. For imaging, MRI and CT are recommended to use for the diagnosis of ischemic diseases.

## 5. Theranostic nanoparticles for vascular injury and restenosis

Vascular injury caused by stenting and restenosis can induce a variety of cardiovascular complications such as thrombosis, MI, and ischemic heart diseases if there is no immediate treatment 111-117. Theranostic nanomaterials developed for vascular injury and restenosis is summarised in Table 4. The regeneration and repair of blood vessels is a crucial way to treat vascular injuries and stenting.^118^, ^119^ The proliferation of smooth muscle cells and endothelial cells plays an important role when the body needs to recover the vessels after injury 120. Polyak et al. 121 designed bovine aortic endothelial cells carrying magnetic iron oxide nanoparticles and adenoviruses that expresses luciferase (Luc). These 290 nm iron oxide nanoparticles could be targeted to an arterial stent under the guidance of an external magnetic field. In *in vivo* experiments, significantly higher expression of Luc was observed in the stent of the magnetic-guided rats than in the non-magnetic control group. Fluorescence imaging of the rat carotid stenting demonstrated that the endothelial cells with iron nanoparticles were able to accumulate in the stented artery segment by exposing the rat to a magnetic field for only 5 minutes. This study showed the potential therapeutic possibilities for vascular regeneration in the stent position.

Anti-inflammatory drugs were also used in theranostic nanomaterials for diagnosis and treatment of in-stent restenosis. Lanza et al. 122 loaded paclitaxel or doxorubicin into perfluorocarbon nanomaterials and conjugated them to the anti-recombinant porcine tissue factor for targeting smooth muscle cells. This nanosystem provided a novel and quantifiable drug delivery system by MRI. Cyrus et al. 123 synthesised a similar perfluorocarbon nanoparticle with α_v_β_3_-target and loaded with rapamycin. The results indicated that α_v_β_3_-targeted nanoparticles could rapidly inhibit the restenosis of New Zealand white (NZW) rabbits and provide reliable MRI imaging within a few minutes after injection. Moreover, Gu et al. 124 developed theranostic layered double hydroxide nanoparticles containing low molecular weight heparin (for treatment) and CdTe quantum dots (for imaging). Results indicated that these nanoparticles were able to target the injured arteries and reduce the injured area of rats.

However, a recent study showed doxorubicin had vascular toxicity and increase the chance of thrombus formation 125. In this case, treating the restenosis patients with doxorubicin could have high risk of side effects. Thus, rapamycin with lower vascular toxicity could be a better choice for the treatment of restenosis. Besides, endothelial cell transplantation is another promising strategy that can be used for treatment. Finally, both perfluorocarbon and iron oxide MRI could be considered as the reliable MRI imaging agent for restenosis.

## 6. Conclusions and Perspectives

In conclusion, in preclinical studies, these multifunctional nanosystems have been successfully applied to cardiovascular diseases, especially atherosclerosis and thrombosis. Currently, the nanomaterials of the core-shell framework are the most popular design for theranostics. Theranostic nanoparticles are basically divided into two components, including a therapeutic agent targeting the pathogenic sites and a diagnostic agent allowing the imaging and tracking of nanoparticles in the body. Drug nanocarriers, most commonly used in the systems of theranostic nanoparticles, typically encapsulate drugs or therapeutic particles within their structures or adsorb the drugs on their surfaces. Additionally, biologics showed effective treatment of cardiovascular diseases.

The most widely used diagnostic imaging agents point to iron oxide nanoparticles, which provide imaging contrast for magnetic resonance imaging (MRI) for various cardiovascular diseases. Besides, perfluorocarbon and gadolinium nanoparticles have emerged to diagnose vascular injury and atherosclerosis via MRI as well as gold nanoparticles to ischemic heart disease via CT. Additionally, imaging agents such as copper sulphide can provide a strong photoacoustic signal for optical imaging of atherosclerosis *in vivo*. Another theranostic system exploits fluorescent dye and polymer to couple with the therapeutic molecules for the *ex vivo* detection and evaluation of therapeutic effects. However, fluorescent agents are unlikely to transfer to clinical trials as the background fluorescence of the human body is much higher than in the cell or mice.

Moreover, by conjugating specific targeting molecules on the surfaces of the nanoparticle, targeted controllable drug release or molecular imaging detection have been achieved. Both antibodies and peptides can provide effective targeting abilities for theranostic nanoparticles. The main biological markers are membranes receptors, cytokines, integrin, thrombin and fibrin. Some other studies showed that folic acid is also a potential target ligand for atherosclerosis. Furthermore, enhanced permeability and retention effect (EPR) is also a critical targeting method for theranostic nanoparticles.

The emergence of theranostic nanoparticles has brought a new dawn for traditional diagnostic and treatment approaches. However, due to the complexity of theranostic nanoparticles, such strategies still face several challenges on the route to clinical translation. The synthesis and optimisation of theranostic nanoparticles need to take into account their effectiveness, long-term stability, purity and sterility, making scaling up the synthesis more challenging. Choosing the right imaging mediums, therapeutic drugs, carrier materials and surface modifiers will be closely related to the efficiency of the theranostic nanoparticles.

The development of a commercial, mass-produced, reliable, repeatable and cost-effective manufacturing method is a challenge for current theranostic nanoparticles. Accelerating the collaboration between researchers and pharmaceutical companies might be a pathway to speed up the development of these agents. Moreover, the intellectual property of theranostic nanoparticles will also be a challenge for the future, as each component could be an independent special patent due to the unique complexity of each agent. Another practical problem is the cost of new drug development. Compared with continuing to use existing diagnosis and treatment methods, new drug development requires a lot of manpower, material resources and time 126. It is worth thinking about how to convince pharmaceutical companies to invest in the research and development of theranostic nanoparticles.

The cellular uptake mechanisms of theranostic nanoparticles in CVDs are not yet fully understood. Some studies suggested its mechanism might be related to the target cells and the particle sizes, but this is still inconclusive and needs to be studied further. Moreover, proper drug release is essential to reduce the biological toxicity of nanoparticles in future preclinical development. The therapeutic window of nanodrugs can sometimes be narrow as the concentration of the nanodrugs that exerts the therapeutic effects is very close to that causes cytotoxicity. Thus, more researches are expected to work on how to safely and efficiently release the therapeutic parts from theranostic nanoparticles to the target areas.

Altogether, theranostic nanoparticles especially those used for CVDs are still in their infancy. Some novel nanoparticles are still in their preclinical stages, while others cannot complete the transition from *in vivo* experiments to clinical trials due to their own limitations. Besides, a mismatch between existing animal models and human diseases could be an obstacle to developing new treatments of diseases 127, 128. Therefore, clinical translation of nanomedicine in general and theranostic nanomaterials in particular might benefit from the establishment of animal models that are more similar to human CVDs.

Overall, nano-therapeutic systems combined with imaging systems promise a more efficient and safer approach to address CVDs. Theranostic nanoparticles with simultaneous treatment and imaging capabilities will play an important role in the diagnosis, treatment and prevention of cardiovascular diseases in the future.

## Figures and Tables

**Table 1:** Theranostic nanoparticles for atherosclerosis.

Carrier nanoparticle material	Imaging agent and imaging modality	Therapeutics/drugs	Target	Binding ligand	Animal model	Ref
High-density lipoprotein (HDL) mimicking nanoparticle	Qdot® 705 ITK™ amino PEG quantum dots Fluorescence	High -density lipoprotein (HDL);	Mitochondria of macrophages	apolipoprotein (apo) A-I mimetic 4F peptide peptide: FAEKFKEAVKDYFAKFWD	Sprague-Dawley rats	60
HDL nanoparticle	Iron oxide - MRI	High-density lipoproteins (HDLs)	N/A	N/A	N/A	20
Perfluorocarbon nanoparticles	Perfluorocarbon - MRI	fumagillin	Alpha-v beta-3 Integrin	lecithin-peptidomimetic vitronectin antagonist	Cholesterol-fed rabbits	34
Iron oxide nanoparticles	Iron oxide - - MRI	prostacycline (PGI2)	N/A	N/A	N/A	26
Iron oxide nanoparticles	Iron oxide - - MRI	CeO2	N/A	N/A	N/A	27, 28
Layered double hydroxide Nanocomposites	Iron oxide - - MRI	CeO2	N/A	N/A	N/A	32
Hybrid lipid nanoparticles (LiLa)	FITC-Fluorescence/Gadolinlium - MRI	Rosiglitazone (Rosi)/ Paclitaxel (PAX)/ tamoxifen (TAM)	Macrophages of the M1 inflammatory phenotype	phosphatidylserine /Oxidized cholesterol ester derivativecholesterol-9-carboxynonanoate (9-CCN)	ApoE knockout mice	71
Carboxyfluorescein nanoparticles	Cy 7 - Fluorescence	Hirulog	Fibrin	CREKA	ApoE knockout mice	62
Simian virus 40 based nanoparticles	Quantum dots 800 near-infrared (NIR)/fluorescence	Hirulog	P32 protein onmacrophages	CGNKRTRGC	ApoE knockout mice	61
Iron oxide nanoparticles	AF750 - NIR fluorescence	meso -tetra(m -hydroxyphenyl)chlorin (phototoxicity)/Gold	Macrophage dextran receptors	Dextran	C57/BL6 mice	69, 70
Selenium nanoparticles	Rose Bengal/ glutathione Fluorescence	Selenium	CD44/FR-β	Hyaluronic acid and folic acid	N/A	56
Deoxycholic acid nanoparticles	Chlorin e6 (Ce6) -Fluorescence	Chlorin e6 (Ce6)	Macrophage dextran receptors	Dextran	N/A	57
Hyaluronic acid nanoparticles	Curcumin - Fluorescence	Curcumin	CD44	Oligomeric hyaluronic acid	N/A	58
Carbon nanotube	Cy5.5 - NIR fluorescence	Photothermal Ablation	N/A	N/A	Carotid‐ligated friend leukemia virus B (FVB) mice	59
Gold nanorods	Gold- CT	Au NIR irradiation	N/A	N/A	Apo E knockout	66
Copper sulfide nanoparticles	Copper sulfide- NIR	Photothermal activation	TRPV1	TRPV1 antibody	Apo E knockout	68

**Table 2:** Theranostic nanoparticles for thrombotic diseases.

Carrier nanoparticle material	Imaging agent and Imaging modality	Therapeutics/drugs	Target markers	Binding ligand	Ref.
Iron oxide nanoparticles	Iron oxide - MRI	rtPA	N/A	N/A	77
Iron oxide nanoparticles	Iron oxide - SPECT/CT	rtPA	N/A	N/A	78
Iron oxide nanoparticles	Iron oxide - MRI	rtPA	Platelet membrane glycoprotein GP IIb//IIIa	RGD peptides	79
Iron oxide nanoparticles	Iron oxide - MRI/ VT680 - Fluorescence	rtPA	Activated factor XIII	Activated factor XIII sensitive peptide (FXIIIa) GNQEQVSPLTLLKC	85
Perfluorocarbon polymer nanoparticles	Perfluorocarbon - MRI	PPACK	Thrombin	PPACK	88-90
borylbenzyl carbonate nanoparticles	IR780/IR820 - NIR	hydroxybenzyl alcohol (HBA)	Fibrin	GPRPP- pentapeptide	91, 92

**Table 3:** Theranostic nanoparticles for myocardial infarction, ischemic heart and ischemic injury

Carrier nanoparticles material	Imaging agent and Imaging modality	Therapeutics/ drugs	Target	Binding ligand	Disease	Ref.
Gold nanoparticles (AuNPs)	Cy 5 -Fluorescence	DNAzyme	Pro-inflammatory cytokine tumor necrosis factor-a gene (TNF-a gene)	TNF-a mRNA: GGACACCAUGAGCAC	Myocardial infarction	101
Iron oxide nanoparticles	Iron oxide - PET, CT & MRI	Mitochondrial transplantation	N/A	N/A	Ischemic Heart	107
Copolyoxalate polymer nanoparticles	Rubrene - Fluorescence	4-AN, HPOX	H_2_O_2_	HPOX	Ischemia- reperfusion injury	110
Iron oxide nanoparticles	Iron oxide - MRI	Stem cell	CD45-exogenous bonemarrow-derived stem cells; endogenous CD34-positive cells to injuredcardiomyocytes	Anti-CD45 and anti- myosin light chain (MLC) antibodies; magnetic attraction	Injured cardiomyocytes	106

**Table 4:** Theranostic nanoparticles for injured arteries.

Carrier Nanoparticle material	Imaging agent and imaging modality	Therapeutics/drugs	Target	Binding ligand	Disease	Ref.
Iron oxide nanoparticles	Iron oxide- MRI	Endothelial cell	Arterial stent	Magnetic target	Injured arteries after stent, lack of reendothelization	121
Perfluorocarbon nanomaterials	Perfluorocarbon - MRI	Doxorubicin and paclitaxel	Smooth muscle cell membranes	Tissue factor	In-stent restenosis	122
Perfluorocarbon nanoparticles	Perfluorocarbon - MRI	Rapamycin	Vitronectin on the injured vessel wall	avb3-integrin	Vascular injury; in-stent restenosis	123
